# Astrocytic GABA transporter activity modulates excitatory neurotransmission

**DOI:** 10.1038/ncomms13572

**Published:** 2016-11-25

**Authors:** Kim Boddum, Thomas P. Jensen, Vincent Magloire, Uffe Kristiansen, Dmitri A. Rusakov, Ivan Pavlov, Matthew C. Walker

**Affiliations:** 1 Department of Clinical and Experimental Epilepsy, UCL Institute of Neurology, University College London, Queen Square, London WC1N 3BG, UK; 2 Faculty of Health and Medical Sciences, Department of Drug Design and Pharmacology, University of Copenhagen, Universitetsparken 2, 2100 Copenhagen, Denmark

## Abstract

Astrocytes are ideally placed to detect and respond to network activity. They express ionotropic and metabotropic receptors, and can release gliotransmitters. Astrocytes also express transporters that regulate the extracellular concentration of neurotransmitters. Here we report a previously unrecognized role for the astrocytic GABA transporter, GAT-3. GAT-3 activity results in a rise in astrocytic Na^+^ concentrations and a consequent increase in astrocytic Ca^2+^ through Na^+^/Ca^2+^ exchange. This leads to the release of ATP/adenosine by astrocytes, which then diffusely inhibits neuronal glutamate release via activation of presynaptic adenosine receptors. Through this mechanism, increases in astrocytic GAT-3 activity due to GABA released from interneurons contribute to 'diffuse' heterosynaptic depression. This provides a mechanism for homeostatic regulation of excitatory transmission in the hippocampus.

## Abstract (web summary)

Astrocytes monitor and regulate both excitatory and inhibitory synaptic activity. Here, the authors identify a novel form of neuronal-glia communication, by which astrocytes detect rises in GABA via the GABA transporter GAT-3; this results in adenosine release that acts presynaptically to inhibit neural glutamatergic signalling.

## Graphical abstract

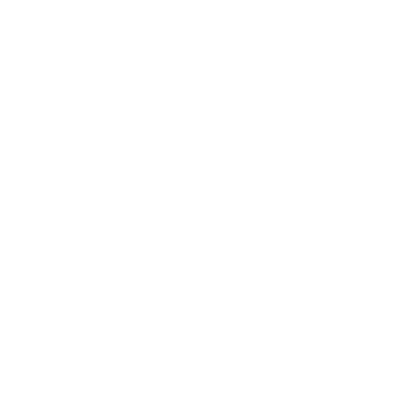

γ-aminobutyric acid (GABA), the main inhibitory transmitter in the brain, binds to post-synaptic ionotropic and metabotropic GABA receptors, and consequently modifies neuronal responses to excitatory inputs by reducing cell excitability. In the hippocampus, GABA is mainly released by interneurons that are recruited through feed-forward or feed-back circuits. This regulates not only network excitability but also the temporal precision of neuronal integration and input discrimination^1^,^2^,^3^. The precision of afferent input is further increased by a complimentary form of inhibition, heterosynaptic depression, in which activation of one afferent pathway depresses the target cell's response to a second pathway. It has long been recognized that heterosynaptic depression accompanies long-term potentiation in the Schaffer collateral pathway^4^. NMDA receptor-dependent release of adenosine contributes to this depression, possibly through the recruitment of interneurons paralleled by Ca^2+^ rises in local astrocytes^5^,^6^. Are these two processes, interneuronal recruitment and astrocyte Ca^2+^ rises, causally related?

Activation of interneurons prompts synaptic GABA release and elevates the extracellular GABA concentration, which is in turn regulated by GABA transporters^7^. Four subtypes of GABA transporters (GATs) have been identified in rat and human: GAT-1, 2, 3 and betaine GABA transporter (corresponding in mice to GAT-1, 3, 4 and GAT-2, respectively)^8^. GAT-1 is primarily responsible for neuronal GABA uptake, and predominantly regulates the GABA concentration detected by pyramidal cells. The role of GAT-3, which is mainly expressed in astrocytes^9^, is less clear but it may play a part in regulating the extracellular GABA detected by interneurons^10^ and be necessary for extracellular GABA regulation during excessive GABA release^11^, such as can occur during periods of elevated network activity.

Here we report a previously unrecognized GABA receptor-independent mechanism through which GABA release from interneurons suppresses glutamatergic signalling in the hippocampus. This novel form of inhibitory GABA action depends on astrocytic GAT-3 activation. Increases in GAT-3 activity result in astrocytic Na^+^ accumulation and a consequent increase in astrocytic Ca^2+^ through Na^+^/Ca^2+^ exchange, leading to the astrocytic release of ATP/adenosine. The resultant rise in extracellular adenosine inhibits glutamate release through presynaptic adenosine receptors. This form of inhibition contributes to the detection and homeostatic regulation of network activity by astrocytes.

## Results

### Activation of GAT-3 inhibits excitatory transmission

**Figure 1:**
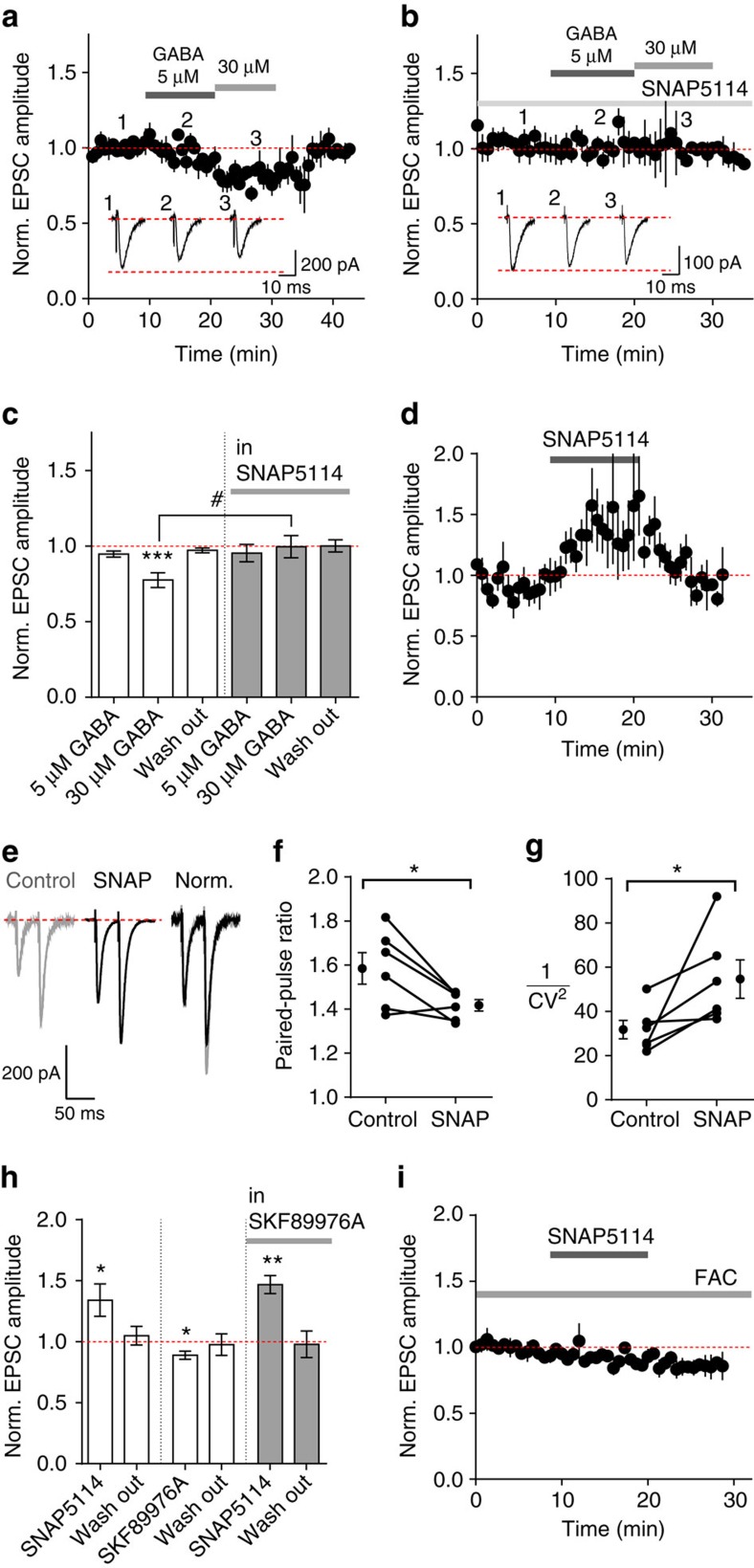
GABA inhibits glutamatergic signalling in a GABA receptor-independent manner through GAT-3 activation. (**a**,**b**) The effect of GABA on EPSC amplitude (*n*=7 cells from 2 animals for 5 μM GABA, *n*=4 cells from 2 animals for 30 μM GABA) is prevented by pre-incubation in the GAT-3 inhibitor, SNAP5114 (100 μM; *n*=6 cells from 3 animals). *Inserts*: Ten EPSCs averaged at indicated time points. (**c**) Summary data for (**a**) and (**b**). (**d**) SNAP5114 (100 μM, *n*=7 cells from 6 animals) increases EPSC amplitude. (**e**) Representative traces of EPSCs evoked in response to paired-pulse stimulation: SNAP5114 decreases the paired-pulse ratio (**f**) and increases 1/CV^2^ (**g**) (*n*=6 cells from 3 animals). (**h**) Summary of normalized EPSC amplitude changes in the presence of GAT inhibitors (SNAP5114: *n*=7 cells from 6 animals; SKF89976A 30 μM: *n*=6 cells from 3 animals; SNAP5114 in slices pre-incubated in SKF89976A: *n*=6 cells from 3 animals). (**i**) Fluoroacetate (FAC; 5 mM) abolishes SNAP5114-induced potentiation of EPSCs (*n*=6 cells from 2 animals). (FAC also causes a slow rundown of EPSCs—possibly reflecting astroglia's role in stable synaptic neurotransmission). Error bars represent s.e.m.'s *P<0.05, **P<0.01, ***P<0.001 (paired *t*-test compared with baseline); ^#^*P*<0.05 (unpaired *t*-test); Norm., normalized.

Whole-cell patch-clamp recordings from CA1 pyramidal neurons in acute hippocampal slices were performed in the presence of GABA_A_-, GABA_B_- and NMDA-receptor antagonists to isolate AMPA receptor-mediated excitatory postsynaptic currents (EPSCs) evoked by electrical stimulation of Schaffer collaterals. Surprisingly, despite the presence of GABA receptor antagonists, application of 30 μM GABA reversibly decreased EPSC amplitudes by 22.5±4.9% (*P*=0.006; *n*=6; Fig. 1a,c). Since a rise in the extracellular GABA concentration also increases GABA transporter activity^12^, we tested the effects of GABA transporter blockers. The glial GABA transporter, GAT-3 inhibitor SNAP5114 (100 μM) prevented the GABA-induced reduction of EPSC amplitude (Fig. 1b,c, *P*=0.031). Moreover, SNAP5114 without exogenously applied GABA increased the EPSC amplitude by 34.0±13.3% (*n*=7; *P*=0.04; Fig. 1d,h), indicating that constitutive GAT-3 activity suppresses EPSCs. SNAP5114 also significantly decreased the paired-pulse ratio and increased 1/CV^2^ of EPSCs (Fig. 1e–g; *P*=0.03; *n*=6) suggesting a presynaptic site of action.

In contrast to the effects of SNAP5114, blocking the primarily neuronal GABA transporter GAT-1 with SKF89976A (30 μM) decreased the EPSC amplitude by 11.2±3.4% of control (*n*=6; *P*=0.02; Fig. 1h). There was also a trend for SKF89976A to potentiate the effect of SNAP5114 (Fig. 1h). These effects are consistent with enhanced GAT-3-mediated activity when neuronal GABA uptake is blocked (it is, however, difficult to directly compare the effects of exogenous GABA application and transporter inhibition, as it is unknown to what extent extracellular GABA concentration increases in local micro-domains as a consequence of transporter inhibition).

Since GAT-3 is predominantly expressed in astrocytes, we asked whether the effects we observed were mediated by astrocytes. We, therefore, tested whether inhibiting astrocytic metabolism with fluoroacetate (FAC, 5 mM) prevented SNAP5114-induced enhancement of EPSCs. In the presence of FAC, SNAP5114 had no effect on EPSC amplitude (Fig. 1i, *P*=0.65; paired *t*-test, *n*=6), confirming an astrocyte-specific locus of SNAP5114's action.

**Figure 2:**
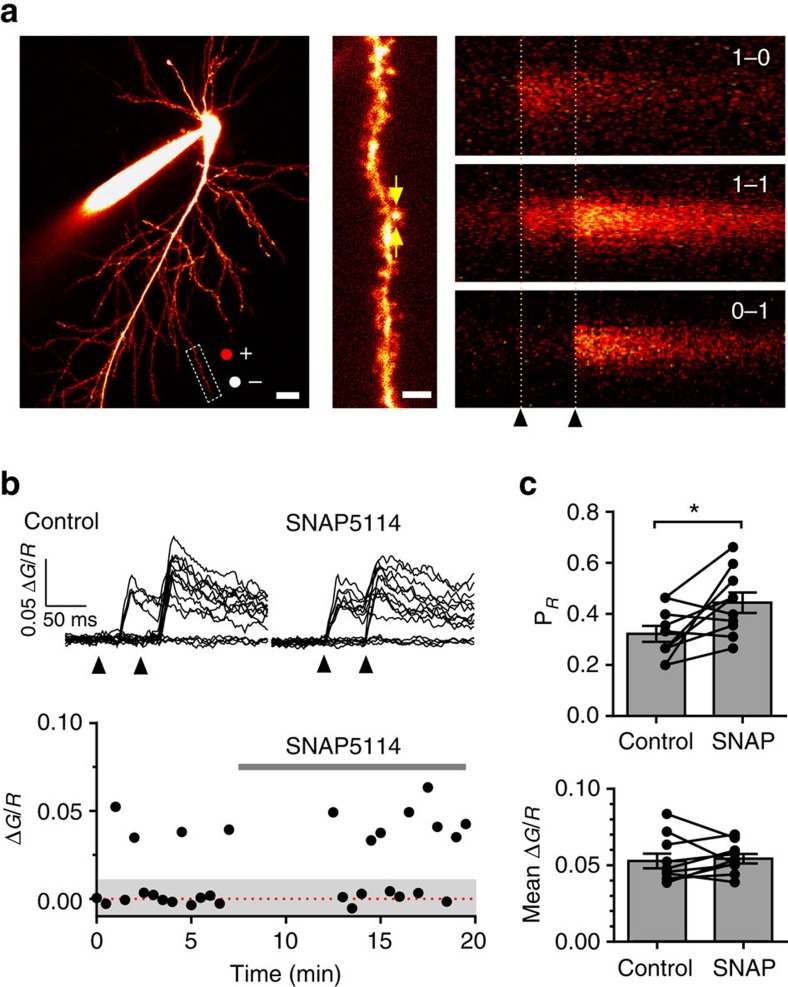
GAT-3 inhibition increases release probability at individual CA3-CA1 synapses. (**a**) Left: Example of recorded CA1 pyramidal neuron (Alexa 594 channel; scale bar, 20 μm). Stimulating pipette locations marked by red and white dots (positive and negative poles). Dashed rectangle indicates a dendritic fragment of interest. Magnified image of the dendrite (Centre; scale bar, 4 μm) shows the position of a line-scan (arrows). Right: Line-scans of the fragment shown in Fluo-2 MA channel. Three typical cases of stochastic responses to paired stimuli (dotted lines) are shown (0 and 1 indicate signal failures and successes, respectively). (**b**) Top: Ca^2+^ fluorescence time course traces (recorded as in a) before and after SNAP application (arrow heads: stimulus onsets). Bottom: Amplitudes of first Ca^2+^ responses in the same experiment (grey shade: the range of failure, twice the baseline noise s.d.). (**c**) Change in release probability (*upper*) and Ca^2+^ Δ*G*/*R* average amplitude of successful responses (*lower*) in response to SNAP5114 (*n*=10 cells from 10 animals). Error bars represent s.e.m.'s; *P<0.05 (paired *t*-test).

To confirm that GAT-3 inhibition resulted in a presynaptic enhancement of excitatory transmission, we used optical quantal analysis of postsynaptic Ca^2+^ responses in CA1 pyramidal cells^13^. By documenting successes and failures of Ca^2+^ rises in individual dendritic spines in response to electrical stimulation of presynaptic fibers, we directly measured the impact of SNAP5114 (and, therefore, tonic GAT-3 activation) on release probability (P_*r*_) and the post-synaptic Ca^2+^ signal (Fig. 2). SNAP5114 increased release probability from 0.32±0.03 to 0.44±0.04 (*n*=10, *P*=0.027), but had no effect on the magnitude of rises in post-synaptic Ca^2+^-dependent fluorescence (*n*=10, *P*=0.72, Fig. 2b,c), indicating a predominant presynaptic effect.

**Figure 3:**
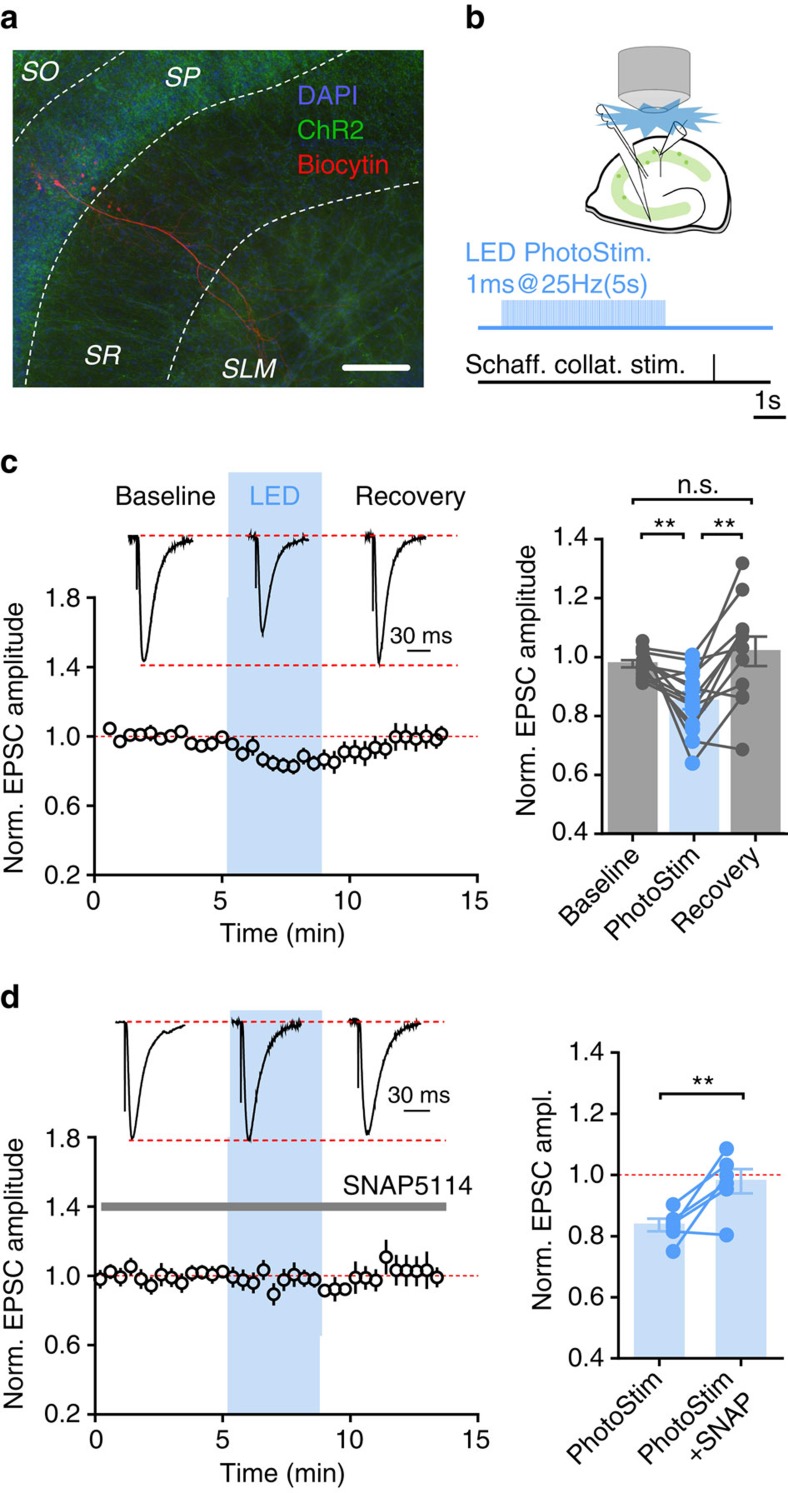
Interneuronal activity suppresses glutamatergic neurotransmission via a GAT-dependent mechanism. (**a**) Hippocampal CA1 region of a mouse with Cre-dependent expression of the EYFP-tagged ChR2 (green) in PV^+^ interneurons. Pyramidal neuron filled with biocytin (red) during a whole-cell patch-clamp recording (scale bar, 100 μm). (**b**) EPSCs were evoked with or without preceding 5 s train of photostimulation of PV^+^ interneurons. (**c**) Selective photo-activation of GABAergic interneurons transiently suppressed EPSC amplitude (*n*=14 cells from 9 animals; left: time course; inset: representative, normalized sample traces; right: summary plot with individual data points). (**d**) SNAP5114 prevents photostimulation-induced EPSC depression (*n*=6 cells from 4 animals; left: time course; inset: representative, normalized sample traces; right: summary plot with individual data points). Error bars represent s.e.m.'s, **P<0.02 (paired *t*-test); n.s.—non-significant.

To further test whether endogenously released GABA from interneurons affects glutamatergic neurotransmission through this mechanism, we optogenetically activated interneurons in hippocampal slices from mice expressing channelrhodopsin-2 (ChR2) in parvalbumin-positive cells. Trains of 470 nm light pulses in the presence of GABA receptor antagonists significantly decreased EPSC amplitudes (by 16.4±2.1%; *n*=14; *P*=0.0024; Fig. 3a–c). This effect on EPSC amplitude reversed once photostimulation was stopped (Fig. 3c) and was occluded by blocking GAT-3 with SNAP5114 (Fig. 3d; *P*=0.017; *n*=6).

### GAT-3 activity evokes astrocytic Ca^2+^ signalling

**Figure 4:**
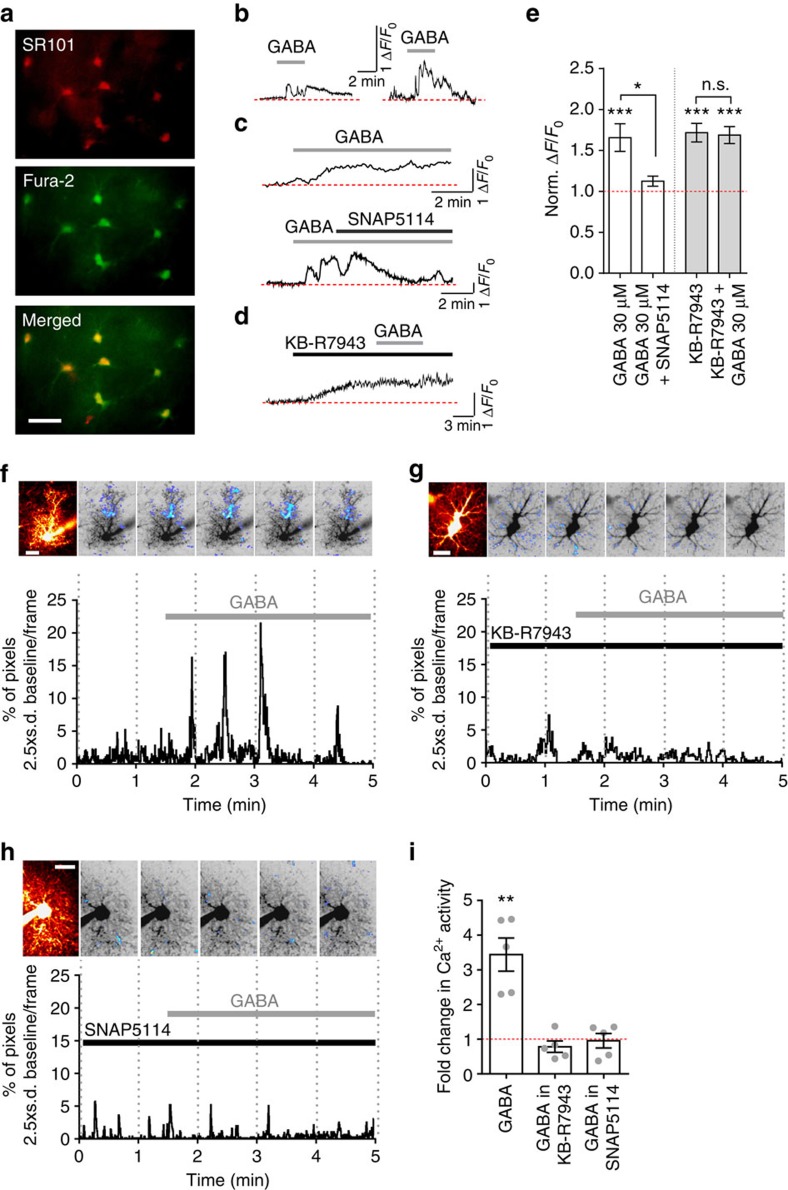
GABA induces GAT-3-dependent astrocytic Ca^2+^ signalling. (**a**) Representative image of astrocytes co-loaded with the astrocytic marker SR101 and Ca^2+^ indicator Fura-2. Scale bar, 20 μm. (**b**–**d**) Representative traces demonstrating: 30 μM GABA-induced changes in astrocytic Ca^2+^ level (**b**); curtailment of GABA-induced increase in Ca^2+^ fluorescence by SNAP5114 (**c**); occlusion of GABA action on intracellular Ca^2+^ by 50 μM KB-R7943 (**d**). (Red dotted line—average baseline fluorescence before drug application) (**e**) Summary plot shows averaged normalized fluorescence signal reflecting the Ca^2+^ rise induced by exogenous GABA (*n*=8 cells from 2 animals), its reversal by SNAP5114 (*n*=8 cells from 2 animals), and the lack of response to GABA application in the presence of KB-R7943 (*n*=8 cells from 2 animals). Data for GABA+SNAP5114 and KB-R7943+GABA were obtained from consecutive applications of either SNAP5114 or GABA, correspondingly, in the recorded cells. (**f**–**h**) Ca^2+^ imaging in astrocytic processes in response to 30 μM GABA application alone (**f**) and in the presence of either 50 μM KB-R7943 (**g**), or 100 μM SNAP5114 (**h**). Patched astrocytes (Alexa 594 channel; scale bars, 20 μm) and thresholded (+2.5 s.d. of the baseline) imaging raster scans (Fluo-2 MA channel) summed for each minute of recordings are shown above Ca^2+^ signalling activity traces. (**i**) Summary plot showing changes in Ca^2+^ activity in response to GABA application (*n*=5 cells from 5 animals for GABA wash-on and GABA in KB-R7943; *n*=5 cells from 4 animals for GABA in SNAP5114). Error bars represent s.e.m.'s *P<0.05, **P<0.01, ***P<0.001 (paired *t*-test compared with baseline); n.s.—non-significant, paired *t*-test.

Activation of GABA uptake would be expected to result in accumulation of intracellular Na^+^ due to GABA:Na^+^ co-transport by GAT-3. This could mechanistically alter astrocyte Ca^2+^ dynamics via the Na^+^/Ca^2+^ exchanger and consequently lead to Ca^2+^-dependent Ca^2+^ release from internal stores^14^,^15^. To test this, we first employed a wide-field imaging approach to determine the effect of exogenously applied GABA on Ca^2+^ dynamics in astrocytes identified by Sulforhodamine 101 counterstaining (Fig. 4a). In all, 30 μM GABA led to an increase in the fluorescence intensity of the Ca^2+^-sensitive dye Fura-2 (by 65.6±16.9%; *P*=0.006; *n*=8), which was suppressed by SNAP5114 (Fig. 4b,c,e; *n*=8, *P*=0.026). This effect is not due to a non-specific inhibition by SNAP5114 of astrocytic Ca^2+^ transients, as activation of the astrocytic metabotropic glutamate receptor with APCD can still elicit Ca^2+^ rises in the presence of SNAP5114 (Supplementary Fig. 1). This also indicates that the increase in the Fura-2 fluorescence intensity by 30 μM GABA is independent from that mediated by APCD.

Activating GAT-3 with the relatively low affinity substrate, β-alanine (1 mM) (ref. 16), which prevents GABA uptake without abolishing transporter action, also resulted in an astrocytic Ca^2+^ rise (Supplementary Fig. 2a). This, however, was considerably smaller than the Ca^2+^ rise observed in response to 30 μM GABA application (c.f. Fig. 4e) and, similar to the application of a low GABA concentration, did not result in a pronounced depression of EPSC amplitude (Supplementary Fig. 2b). However, a higher concentration of β-alanine (2 mM), as would be predicted for a low affinity substrate, resulted in a significant reduction of EPSC amplitude by 17±2% (*n*=3, *P*=0.0004, Supplementary Fig. 2c).

**Figure 5:**
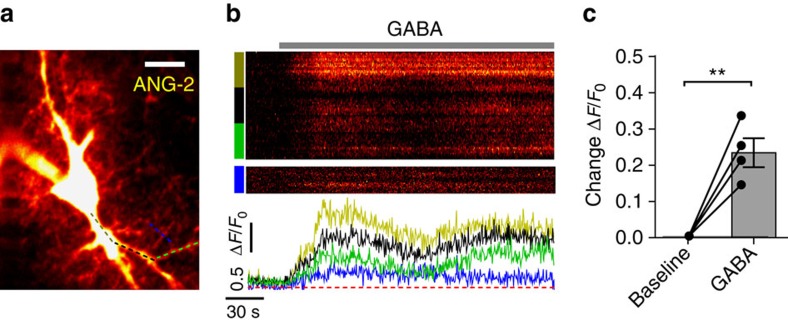
GABA induces Na^+^ rise in astrocytic processes. (**a**) Example image of an astrocyte patch loaded with the Na^+^ indicator ANG-2, dashed colour-coded lines indicate analysed regions in (**b**). Scale bar, 10 μm. (**b**) Upper section: XY-T image displaying ANG-2 fluorescence intensity vs time, separated section shows Na^+^ signal in fine processes; contrast and brightness were altered to facilitate visualization of the weaker signal. Lower section: colour coded Δ*F*/*F*_0_ line plots match regions indicated by coloured bars/dashed lines in upper section and (**a**) respectively. (**c**) Summary plot of peak Na^+^ indicator response to 30 μM GABA application in fine processes of imaged astrocytes (*n*=4 cells from 4 animals). Error bars represent s.e.m.'s, **P<0.01 (paired *t*-test).

Blocking the Na^+^/Ca^2+^ exchanger using KB-R7943 increased the intracellular Ca^2+^ (*n*=8, *P*=0.0004) and occluded the effect of GABA on intracellular Ca^2+^ levels (Fig. 4d,e; *P*=0.2; *n*=8). In line with this, KB-R7943 also reduced EPSC amplitude to 84.6±3.7% of baseline (*n*=4, *P*=0.002). Furthermore, application of 30 μM GABA in KB-R7943 had no effect on the EPSC amplitude (96.7±2.9% of response in KB-R7943, *n*=5, *P*=0.32; GABA wash-out 97.4±4.3%, *n*=5, *P*=0.9). Recent studies of astrocyte–synapse interactions suggest that modulation of synaptic neurotransmission could rely on highly compartmentalized Ca^2+^ dynamics in astrocytic processes^17^,^18^. We, therefore, asked whether GABA-induced Ca^2+^ rises occurred in astrocytic processes or were restricted to the astrocytic soma. In order to understand astrocytic Ca^2+^ dynamics at the subcellular level, we used two-photon excitation imaging of astrocytes, individually loaded with Fluo-2 MA in whole-cell mode (Fig. 4f–i). We found that, in addition to overall somatic Ca^2+^ increases in Fura-2 loaded cells (Fig. 4a), GABA application increased local Ca^2+^ transients in fine astrocytic processes (*P*=0.007, *n*=5), and that these were also significantly suppressed by KB-R7943 and SNAP5114 (Fig. 4f–i; in these tests, Ca^2+^ increases in the soma, but not in astrocytic processes, were likely to be suppressed due to the proximity of the dialysing pipette tip^19^). Furthermore, two-photon excitation Na^+^ imaging with the indicator ANG-2 (ref. 20) confirmed detectable GABA-induced Na^+^ rises in astrocytic processes in response to GABA application (Fig. 5, *n*=4, *P*=0.0003).

**Figure 6:**
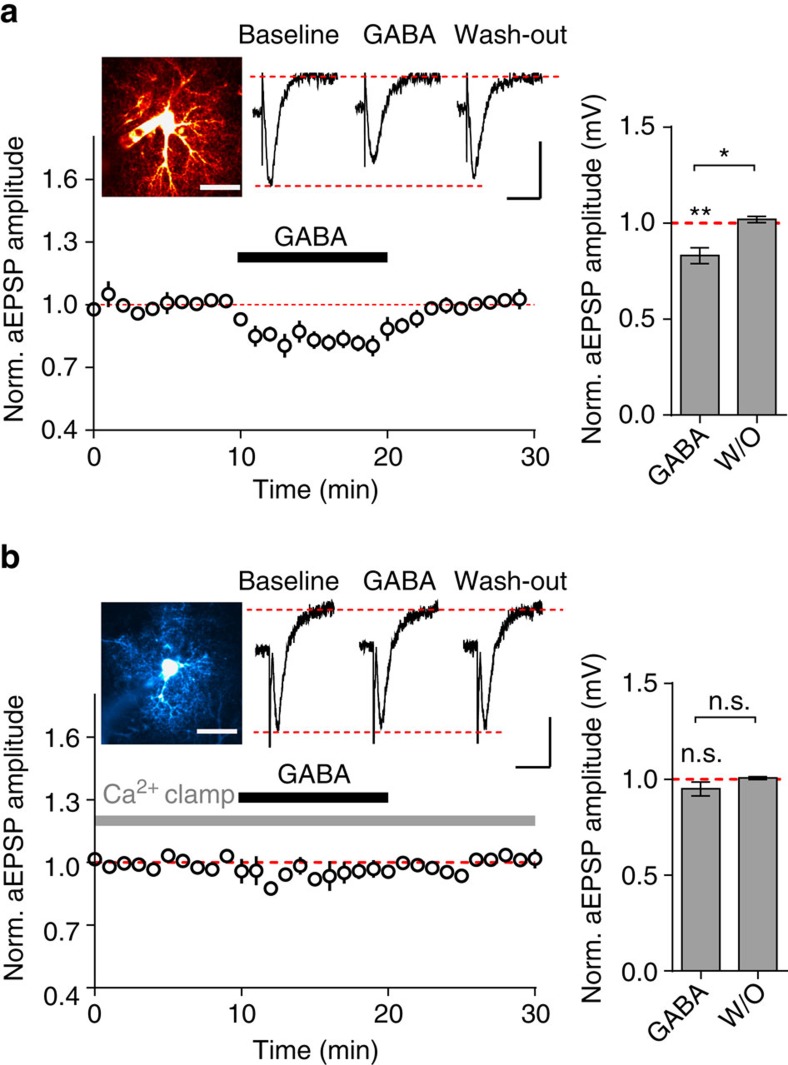
Astrocytic Ca^2+^ clamp prevents suppression of field EPSPs by GABA. (**a**) Time course (left) and a summary plot (right) of the effects of GABA on normalized field EPSPs recorded through CA1 passive astrocytes (aEPSPs; *n*=6 cells from 5 animals). Inset: An example of a patched astrocyte loaded with Alexa 594 and sample traces of aEPSPs (conditions indicated above traces). Image scale bar, 20 μm, aEPSP scale bars: vertical 0.2 mV, horizontal 30 ms. (**b**) Single cell examples and summary plots (*n*=5 cells from 4 animals) of GABA effect on aEPSPs recorded though an astrocyte filled with an internal solution buffered to a nominal Ca^2+^ concentration of 50–80 nM (Ca^2+^ clamp). Layout as in **a**; vertical aEPSP scale bar, 0.3 mV, horizontal 30 ms; image scale bar, 20 μm; error bars represent s.e.m.'s; *P<0.05, **P<0.01, n.s.—non-significant (paired *t*-test).

To further demonstrate that GAT-3-mediated astrocyte Ca^2+^ rises are indeed causal to the suppression of excitatory transmission, we obtained whole-cell current-clamp recordings from CA1 astrocytes, and were thereby able to combine internal Ca^2+^ concentration clamp with local field excitatory postsynaptic potential (fEPSP) monitoring^21^. By including known Ca^2+^ and Ca^2+^ buffer concentrations within the internal solution, one can clamp [Ca^2+^] within an astrocyte to baseline levels (50–80 nM) while taking advantage of the low membrane resistance of passive astrocytes to measure the fEPSP through the patch pipette (aEPSP). In the absence of any additional Ca^2+^ buffer, GABA significantly suppressed the aEPSP (to 83.1±4.1% of baseline, *P*=0.01; *n*=6, Fig. 6a), consistent with Fig. 1a. In contrast, buffering the internal Ca^2+^ concentration to ∼50–80 nM with intracellular Ca^2+^ buffers prevented this decrease in aEPSP amplitude (95.0±3.5% of baseline, *P*=0.24, *n*=5, Fig. 6b). Using a repeat measures ANOVA (between-subject factor: presence of buffer; within-subject factors: GABA and interaction of GABA with presence of buffer), there was a significant effect of the interaction of GABA with presence of buffer (*F*_1,9_=5.39, *P*=0.045). This implies that changes in the internal astrocytic Ca^2+^ concentration modify the effect of GABA on aEPSP amplitude.

### GAT-3 activity and A_1_ receptor activation

**Figure 7:**
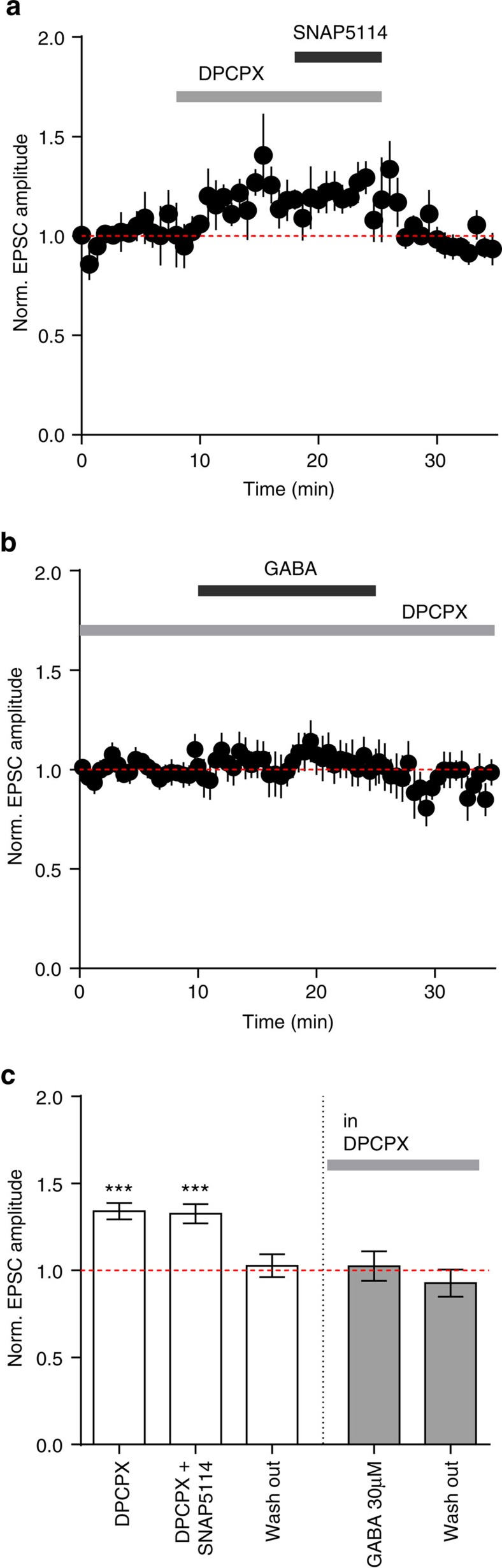
GAT-3-mediated EPSC inhibition depends on astrocytic Ca^2+^ signalling and ATP/adenosine release. (**a**) A_1_ antagonist DPCPX (10 μM) increases amplitude of EPSCs and occludes the effect of SNAP5114 (*n*=6 cells from 4 animals). (**b**) In all, 30 μM GABA in the presence of DPCPX does not alter EPSC amplitude (*n*=7 cells from 4 animals). (**c**) Summary plot showing the effects of DPCPX alone, and SNAP5114 and GABA in the presence of DPCPX on normalized EPSC amplitudes. Error bars represent s.e.m.'s, *** *P*<0.001 (paired *t*-test compared with baseline).

Astrocytes display Ca^2+^-mediated adenosine and vesicular ATP release^22^,^23^; extracellular ATP is then rapidly degraded to adenosine by ecto-ATPases^24^. Therefore, we sought to determine if presynaptic adenosine A_1_ receptors mediate the GAT-3-dependent, presynaptic decrease in glutamate release. Blocking A_1_ receptors with 8-cyclopentyl-1,3-dipropylxanthine (DPCPX; 10 μM) increased the evoked EPSC amplitude (34.1±16.6%; *n*=6; *P*=0.001; Fig. 7a,c), similar to that induced by SNAP5114. Moreover, DPCPX occluded the effect of SNAP5114 on EPSC amplitudes (*n*=6; *P*=0.8; Fig. 7a,c). Pre-treatment with DPCPX also prevented the effects of GABA on EPSC amplitudes (*n*=7; *P*=0.8, Fig. 7b,c).

### GAT-3 activation and heterosynaptic depression

**Figure 8:**
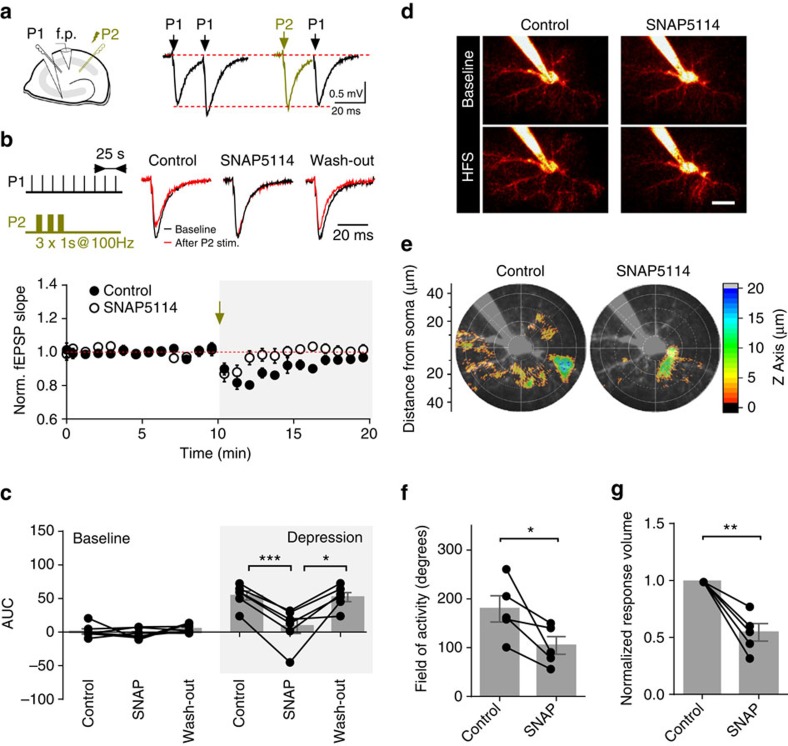
Inhibition of glial GABA uptake reduces high-frequency-induced heterosynaptic depression and astrocytic Ca^2+^ response. (**a**) Experimental schematic and sample field EPSPs (fEPSPs) evoked in response to paired-pulse stimulation used to establish pathway independence (P1: pathway one; P2: pathway two). (**b**) Tetanic stimulation (3 × 1 s at 100 Hz) of P2 pathway causes transient heterosynaptic depression in P1 pathway, which is reduced in the presence of SNAP5114 (*n*=6 slices from 5 animals). Amplitude of traces is normalized. (**c**) The time course of fEPSP slope changes was integrated 10 min before and after tetanization to quantify the amount of depression in each slice in control, after application of SNAP5114 and following SNAP5114 wash-out (*n*=6 slices from 5 animals). (**d**) Sample images of Fluo-2 MA fluorescence in CA1 passive astrocytes at baseline (top) and at the peak of the Ca^2+^ response induced by high-frequency tetanic stimulation (1 s at 100 Hz; bottom) in control conditions and in the presence of 100 μM SNAP5114 (scale bar, 20 μm). Note the reduction in the Ca^2+^ response in the presence of SNAP5114. (**e**) Representative radial plots of 3D reconstructed Ca^2+^ responsive volumes derived from the recording in **d**. Responsive volume in control conditions (left) and in the presence of SNAP5114 (right). Colour scale indicates the depth of the astrocyte over which activity was detected. (**f**) Radial field over which Ca^2+^ responses could be detected after tetanic stimulation is decreased in SNAP5114 (*n*=5 cells from 5 animals). (**g**) The relative change in estimated Ca^2+^ response volume in control conditions and in the presence of SNAP5114 (*n*=5 cells from 5 animals). Individual recordings represented by dots, means displayed by bars, error bars represent s.e.m.'s; *P<0.05; **P<0.01; ***P<0.001 (paired *t*-test); AUC, area under the curve; HFS, high-frequency stimulation.

To understand the role of GAT-3 activation on heterosynaptic depression, we stimulated two distinct Schaffer collateral pathways and recorded fEPSPs in the absence of any neurotransmitter antagonists (Fig. 8a). We intermittently tetanized one pathway (stimulated pathway), and determined the effect of the tetani on the amplitude of the fEPSP in the other pathway (test pathway). The tetani consistently induced depression in the test pathway. This heterosynaptic depression was significantly reduced by inhibiting GAT-3 with SNAP5114 (Fig. 8b,c; *n*=6; *P*=0.0002). In a separate set of experiments, we used two-photon excitation three-dimensional (3D) Ca^2+^ imaging to further confirm that GAT-3 activity strongly influences the spatial spread and amplitude of the Ca^2+^ rise that follows such tetanic stimulation (Fig. 8d–g). Tetanic stimulation results in a large rise in Ca^2+^ indicator fluorescence in the processes of the imaged astrocytes that is significantly reduced by SNAP5114 (Supplementary Fig. 3). However, Ca^2+^ signals measured within one focal plane do not fully represent the extent of Ca^2+^ activity or its changes in all three dimensions of the astrocyte arbor. To better understand such activity, we estimated the spatial extent of Ca^2+^ rises across the 3D astrocytic morphology including the overall volume over which the astrocytic Ca^2+^ response peaks (Fig. 8e). SNAP5114 significantly reduced the total volume and spatial extent of Ca^2+^ rise in astrocytes observed during tetanic stimulation by 45.6±7.7% (*P*=0.004) and 41.8±10.2% (*P*=0.015), respectively (*n*=5, Fig. 8f,g).

## Discussion

Recent work has indicated that astrocytes, which had previously been proposed to have predominantly a supportive role, have crucial functions in determining network excitability, and both respond to and modulate neuronal activity^25^,^26^,^27^. It has been shown that astrocytes detect network activity through metabotropic and ionotropic receptors, and modulate neuronal excitability by the release of gliotransmitters, in particular glutamate, ATP (adenosine), D-serine and neurotrophins^28^. Indeed, astrocytes serve a panoply of homeostatic roles including spatial isolation of synapses, buffering of extracellular potassium and regulation of extracellular neurotransmitter concentrations. However, in contrast to the regulation of extracellular glutamate by astrocytic glutamate transporters^29^, the role of astrocytic GABA transporters in regulating extracellular GABA in the hippocampus remains poorly characterized. Here we show a novel role for astrocyte expressed GABA transporters in the hippocampus to regulate astrocytic release of ATP/adenosine, and so modulate excitatory transmission. Moreover, we show that this process is activated by endogenous GABA release and that it can mediate heterosynaptic depression.

It has previously been suggested that astrocytes express both GABA_A_ and GABA_B_ receptors and that activation of these receptors can result in increases in intracellular astrocyte Ca^2+^ concentrations^30^,^31^,^32^,^33^. The role of astrocyte expressed GABA_A_ receptors is not entirely clear; GABA_A_ receptors activation tends to depolarize astrocytes and can activate voltage-gated Ca^2+^ channels. However, phasic activation of GABA_A_ receptors by endogenous GABA has yet to be shown. Nevertheless, prolonged activation of astrocytic GABA_A_ receptors can affect morphological differentiation of astrocytes during development. Endogenous GABA can, however, also activate astrocytic GABA_B_ receptors resulting in increases in astrocytic Ca^2+^ through release of Ca^2+^ from IP3-sensitive intracellular stores.

In addition to receptor-mediated signalling GABA can affect glial physiology through GABA transporters (for example, ref. 34). GABA transporter expression is cell specific and GAT-3 is predominantly expressed on astrocytes in the hippocampus. It has been previously shown that GAT activation can result in Ca^2+^ rises in astrocytes and that this can contribute to the regulation of vascular tone^14^. Here we have demonstrated that GAT-3 activation results in a rise in astrocytic Na^+^ and Ca^2+^ in astrocyte processes. Moreover, inhibiting Na^+^/Ca^2+^ exchange prevents the GAT-3-dependent astrocyte Ca^2+^ rises. This implies that the increase in astrocyte Na^+^ drives the Ca^2+^ rise, similar to that observed for astrocytes in the developing olfactory bulb^14^; indeed, this may represent a ubiquitous mechanism in the central nervous system. We have shown that such Ca^2+^ rises occur not only in response to exogenously applied GABA but also during synaptic activity. Astrocytes can, therefore, detect extracellular GABA through three separate mechanisms (ionotropic receptors, metabotropic receptors and transporter activity). These may act over different temporal scales and may be regulated differently at distinct stages of development, but a precise functional division is still unclear.

We observed that tonic activity of astrocytic GAT-3 was sufficient to lead to presynaptic inhibition of excitatory transmission, and that increases in extracellular GABA either through exogenous application or increases in interneuron activity could increase this inhibition. Moreover, by using optical quantal analysis of post-synaptic Ca^2+^ transients, we showed that this is through a direct inhibition of presynaptic release probability.

This inhibition of excitatory transmission could be occluded either by clamping the astrocyte Ca^2+^ concentration to baseline levels (50–80 nM) or by inhibiting presynaptic A_1_ adenosine receptors. Altogether, these data imply that the GAT-3-induced Ca^2+^ rises result in increases in extracellular adenosine (through astrocyte ATP and/or adenosine release) that acts on presynaptic A_1_ receptors. Thus, this GAT-3-mediated signalling acts in synergy with the proposed mechanism for astrocyte GABA_B_ receptor mediated inhibition of excitatory transmission^6^. Whether ATP/adenosine is released in this case in a vesicular fashion and involves SNARE machinery^35^ warrants additional investigation. We also do not know whether the network effects of GABA acting on astrocytic signalling are limited to excitatory neurotransmission or whether GABAergic signalling is also modulated (for example, refs 30, 36, 37). Further research is also needed to establish whether the release of other gliotransmitters such as D-serine, purines and glutamate is affected by GAT-3 activity. It is likely that Ca^2+^ rises that are recorded in astroglia in response to different stimuli have highly distinct sources, underlying molecular cascades and spatiotemporal dynamics that may result in different patterns of gliotransmitter release^38^.

We have further shown that during high-frequency stimulation, a protocol that is commonly used to induce Schaffer collateral long-term potentiation, there are rises in astrocyte Ca^2+^ and consequent heterosynaptic depression that are GAT-3 dependent. Importantly, these experiments were performed without any exogenous receptor antagonists, and so demonstrate a counterintuitive phenomenon in which inhibition of glial GABA uptake decreases network inhibition. The contribution of GAT-3 activation to heterosynaptic depression is an unexpected observation, and increases the range of mechanisms by which heterosynaptic depression can occur. Heterosynaptic depression through activation of interneurons and consequent ‘release' of adenosine has long been recognized, but the mechanisms underlying this have only been partially elucidated^5^,^6^. Such heterosynaptic depression permits competitive network activity. This is a computationally powerful process, which enables ‘winner takes all' during synaptic plasticity and promotes sparse coding, so optimizing information processing and storage in the hippocampus. Moreover, GAT-3 activity is sensitive to low concentrations of GABA^16^ making it an ideal detector of neuronal activity, so providing a powerful method for homeostatic network control.

## Methods

### Hippocampal slice preparation

Animal procedures were subject to local ethical approval and adhered to the United Kingdom Animals (Scientific Procedures) Act of 1986. Male Sprague Dawley rats (P19-P26) were sacrificed using an overdose of isoflurane. Animals were kept in groups (5–8 per cage) under standard housing conditions with 12 h light–dark cycle and free access to food pellets and drinking water. After decapitation brains were rapidly removed, hippocampi dissected out and transverse slices were prepared on a Leica VT1200S vibratome (350 μM thick for electrophysiological and two-photon imaging experiments, or 250 μM thick for Fura-2 imaging experiments). The slicing was performed in ice-cold slicing solution that for electrophysiology contained (in mM): 75 sucrose, 87 NaCl, 22 glucose, 2.5 KCl, 7 MgCl_2_, 1.25 NaH_2_PO_4_, 0.5 CaCl_2_ and 25 NaHCO_3_; for Fura-2 imaging the solution contained (in mM): 100 NMDG, 20 NaCl, 22 glucose, 2.5 KCl, 1.3 MgSO_4_, 1 NaH_2_PO_4_, 25 NaHCO_3_, both 315-330 mOsm. For two-photon imaging slicing solution contained (in mM): 60 NaCl, 105 sucrose, 26 NaHCO_3_, 2.5 KCl, 1.25 NaH_2_PO_4_, 7 MgCl_2_, 0.5 CaCl_2_, 11 glucose, 1.3 ascorbic acid and 3 sodium pyruvate. All solutions were bubbled with 95% O_2_ plus 5% CO_2_, pH adjusted to 7.4. Slices were incubated in the oxygenated storage solution at 34 °C for 15 min and then allowed to equilibrate to room temperature for 15 min. Slices used for electrophysiological recordings were then transferred to a continuously oxygenated humid interface chamber and allowed to recover at room temperature for at least 60 min. Recordings were performed in a recording chamber constantly perfused with 32–34 °C oxygenated artificial cerebrospinal fluid (aCSF) solution using a gravity driven perfusion system. aCSF solution contained (in mM): 120 NaCl, 22 glucose, 2.5 KCl, 1.3 MgSO_4_, 1 NaH_2_PO_4_, 25 NaHCO_3_, 1.7 MgCl, 2.5 CaCl_2_, had osmolality of 296 mOsm and equilibrated with 95% O_2_ plus 5% CO_2_. Storage solution had Ca^2+^ concentration reduced to 1 mM. A cut was made between the CA1 and CA3 regions to prevent propagation of recurrent excitation through Schaffer collaterals.

### Electrophysiological recordings

Whole-cell patch-clamp recordings were performed from CA1 pyramidal cells (input resistance 330±70 MΩ) visualized using an infrared differential contrast imaging system. Standard walled borosilicate glass capillaries were used to fabricate recording electrodes with a resistance of 2.5–3.5 MΩ. Intracellular pipette solution contained (in mM): 120 Cs-methanesulfonate, 10 HEPES, 0.2 EGTA, 8 NaCl, 0.2 MgCl_2_, 2 Mg-ATP, 0.3 Na-GTP, 5 QX314-Br, 10 phosphocreatine, pH adjusted to 7.2 and osmolality adjusted to 296 mOsm. Series resistance was monitored throughout experiments using a −5 mV step command. Cells showing a series resistance >20 MΩ, a >20% change in series resistance, or unstable holding current were rejected. EPSCs were evoked by stimulation of Schaffer collaterals with bipolar tungsten electrodes at 0.025 Hz. Paired responses were evoked with 50 ms inter-stimulus intervals.

For astrocyte Ca^2+^ clamp experiments CA1 Stratum radiatum passive astrocytes were patched in whole-cell mode with an internal solution containing (in mM): 135 potassium methanesulfonate, 10 HEPES, 10 di-Tris-Phosphocreatine, 4 MgCl_2_, 4 Na_2_-ATP, 0.4 Na-GTP (pH adjusted to 7.2 using KOH, osmolarity 290–295), and supplemented with the morphological tracer dye Alexa 594 (50 μM). To maintain intracellular free Ca^2+^ concentration at a steady state level of 50–80 nM, the internal solution was supplemented with 0.2 mM BAPTA, 0.45 mM EGTA and 0.14 mM CaCl_2_. Following whole-cell access, astrocytes were identified by criteria previously described^39^ and left for 25 min for the internal solution and Ca^2+^ buffers to equilibrate across the astrocytic arbor. Excitatory synaptic responses were monitored as field potentials recorded through the astrocyte patch-pipette in current clamp mode (aEPSPs) as previously described^21^ and evoked by stimulation of Schaffer collaterals with bipolar tungsten electrodes 200 μm distant to the astrocyte at a rate of 0.033 Hz. Stimulus intensity was normally set at ∼50% of maximum response amplitude.

FEPSPs were recorded using 1.5–2 MΩ resistance glass electrodes filled with aCSF. Stimulation intensity was set to induce half-maximal responses. Heterosynaptic depression in the CA3-CA1 synapses was induced in the absence of glutamate and GABA receptor antagonists by tetanising one of the two independent Schaffer collateral pathways with three consecutive 1 s 100 Hz trains using bipolar tungsten stimulation electrodes. Pathway independence was established at the start of each experiment using paired-pulse stimulation (20 ms inter-pulse interval). Paired responses of each pathway were compared with those evoked by activating the pathways at 20 ms intervals. Ca^2+^ concentration in the aCSF in these experiments was reduced to 1.5 mM to facilitate the induction of heterosynaptic depression. fEPSP slopes were measured to determine changes in the evoked responses. Since heterosynaptic depression lasts minutes (see Fig. 8b), we represented the amount of depression as area under the curve rather than peak depression.

Recordings were obtained using a MultiClamp 700 B amplifier (Axon Instruments, Foster City, CA, USA), filtered at 4 kHz, digitized and sampled through an AD converter Digidata 1550 (Molecular Devices) or NI PCI-6221M (National Instruments) at 10 kHz and stored on a PC. LabView routines (National instruments, Newbury, UK), pClamp10 software (Molecular Devices) or WINWCP (John Dempster, Strathcylde Elecrophysiology Software) were used for data acquisition and off-line analysis (blinded to group). Average EPSC amplitude values were calculated for 5-min periods immediately before drug applications, and at the end of drug wash-in and wash-out periods.

### Optogenetic experiments

*PV::cre mice* (*B6;129P2-Pvalb*^*tm1(cre)Arbr*^/J Jackson laboratory stock number: 008069) were crossed with Ai32 mouse line, which has floxed-stop EYFP-tagged excitatory opsin ChR2 (*B6;129S-Gt(ROSA)26Sor*^tm32(CAG-COP4*H134R/EYFP)Hze^/J Jackson laboratory stock number: 012569), to produce animals with ChR2 expression in parvalbumin positive (PV^+^) interneurons throughout the brain^40^. Animals were kept under standard housing conditions with 12 h light–dark cycle and free access to food pellets and drinking water. Hippocampal slices were prepared from mice of both sexes aged between postnatal day 25 and 50. Littermates of the same sex were housed in groups of 3–5 animals. Wide-field illumination of the CA1 region of the hippocampus was delivered through 20 × water immersion objective (Olympus). Blue light (wavelength 470 nm) was generated using pE-2 LED illumination system (CoolLED); light intensity at the surface of the slice was in the range of 5–7 mW. PV^+^ interneurons were activated using 5 s trains of 1 ms pulses delivered at 25 Hz. To test the effect of GABA released from PV^+^ interneurons on AMPA receptor-mediated currents in the Schaffer collaterals synapses EPSCs were evoked every 12 s either in the absence of photostimulation, or 1,250 ms after the termination of the light train. Ten EPSCs were averaged at the end of each period: 5 min baseline, 3 min photostimulation and 5 min recovery.

### Fura-2 Ca^2+^ imaging

Before Ca^2+^ imaging, slices were incubated with Sulforhodamine 101 (SR101; 5 μM) and the high-affinity Ca^2+^-sensitive dye, Fura-2 AM (5 μM), in aCSF solution for 35 min at 34 °C while bubbled with 95% O_2_ plus 5% CO_2_. Live cell imaging was performed at room temperature using an Olympus BX51WI (USA) microscope, with a 40 × PlanApo objective (NA 0.8). Fluorescent dyes were excited at 340/380 nm (Fura-2) and 570 nm (SR101) with a xenon light source (Polychrome IV or V, TILL Photonics, Germany). Images were recorded with a SensiCam cooled CCD (charge-coupled device) camera (PCO Imaging, Germany) controlled by TILL-visION imaging software (TILL Photonics, Germany). Fluorescent emission was recorded at 0.5–2 Hz from elliptical regions of interest placed over individual astrocytes. The ratio between the measured values of Fura-2 fluorescence (340/380) was background subtracted, normalized to baseline (*F*_0_) and reported as Δ*F*/*F*_0_. Average Ca^2+^ response was calculated for 2-min periods 2 min after drug applications, and at the end of drug wash-out periods. Data for GABA+SNAP5114 and KB-R7943+GABA were normalized to baseline fluorescence before GABA or KB-R7943 application correspondingly.

### Two-photon excitation imaging

We used a Femtonics Femto3D-RC imaging system (Femtonics Femto 3D-RC, Budapest) optically linked to a femtosecond pulse laser MaiTai (SpectraPhysics-Newport) and integrated with patch-clamp electrophysiology (25 × Olympus objective, NA1.05). For imaging of spontaneous and evoked astrocytic Ca^2+^ dynamics, CA1 Stratum radiatum passive astrocytes were patched with the potassium methanesulfonate-based internal solution described above supplemented with the morphological tracer dye Alexa 594 (50 μM) and either the Ca^2+^ indicator Fluo-2 MA (200 μM) or Na^+^ indicator ANG-2 (200 μM).

Following whole-cell access, astrocytes were identified by criteria previously described^39^ and left for 25 min for fluorophores to equilibrate across the astrocytic arbor. For time-lapse imaging of astrocytic Ca^2+^ transients and GABA evoked Na^+^ responses, images were collected in frame scan mode for 5 min at a resolution of∼0.4 μm per pixel and image size appropriate for the shape of the astrocyte, frame rate was ∼3 Hz per time point. For imaging of evoked astrocytic Ca^2+^ responses four-dimensional frame scanning was implemented using a fast piezo-objective positioner of the Femto3D-RC setup. In this mode four focal planes separated by five microns were scanned sequentially for 40 s. Two-dimensional image dimensions and scan rate were preserved by reducing the pixel dwell time. Ca^2+^ responses were generated spontaneously in response to GABA application or evoked by 1 s 100 Hz trains of electrical stimulation using the same parameters and method described above for aEPSP recordings.

### Analysis of spontaneous and evoked Ca^2+^ transients

For analysis of spontaneous Ca^2+^ transients in astrocytic fine processes, Ca^2+^ responses were defined as regions where an increase in ΔG/R >2.5 × s.d. of the baseline occurred. To obtain these data image stacks were opened in ImageJ (Rasband WS, ImageJ, NIH, Bethesda, Maryland, USA, http://rsb.info.nih.gov/ij/, 1997–2008), pixel binned 2 × 2 (x,y), and processed to make a *G*-*G*_min_/R image stack where G_min_ is a minimum intensity projection of the stack in the time domain. This image stack was then median filtered along the time domain to remove shot noise, and 20 frames in which no visually identifiable Ca^2+^ signals occurred were chosen to produce a baseline image stack. Both baseline image stack and complete stack were then imported into MATLAB 2015a and an image corresponding to the mean+2.5 × s.d. of the baseline stack was used as a threshold to segment each frame into active and inactive pixels. Ca^2+^ signalling activity was then expressed as the percentage of the imaged area active (>2.5 × s.d. baseline Δ*G*/*R*) per frame. For comparisons between experimental conditions, the mean area active for 1.5 min before drug application was compared with the mean area active for 1.5 min, 1.5 min following drug wash on.

For 3D analysis of the spread of high-frequency stimulus-evoked astrocytic Ca^2+^ transients amplitude thresholding produced qualitatively inaccurate representations of the spread of the tetanic stimulation-evoked Ca^2+^ signal across the volume imaged. As a result a local cross-correlation approach previously described in ref. 41 (code available from http://labrigger.com/blog/2013/06/13/local-cross-corr-images/) was used to produce an activity heat map from the time lapse image stacks for each focal plane imaged. The heat map images were then thresholded to the top 5% cross-correlation scores to distinguish the locations of active regions within each focal plane. To identify the field of activity around the astrocytes soma the thresholded (binary) images from each focal plane were summed and the radial reslice function in ImageJ (Rasband WS, ImageJ, NIH, Bethesda, Maryland, USA, http://rsb.info.nih.gov/ij/, 1997–2008) was used to identify the total radial coverage around the astrocytes soma where active regions were identified. Estimates of the volume of responsive regions were by the sum of active voxel volumes within each image.

### Postsynaptic Ca^2+^ imaging in CA1 pyramidal neurones

Postsynaptic imaging of Ca^2+^ responses (optical quantal analysis) in CA1 pyramidal cell dendrites and spines was carried out as previously described^13^,^42^. Local, minimal stimulation was provided by bipolar stimulation using a pair of glass stimulating electrodes filled with aCSF. The electrodes were placed parallel to the targeted dendritic branch, at a distance of ∼20–30 μm form the branch. To identify active synapses fast (20 Hz), frame scans of the local dendrites were viewed while three 100-μs square pulses of 2–10 V were delivered with a 25-ms interstimulus interval using a constant voltage isolated stimulator (model DS2A-mkII; Digitimer). This protocol was repeated until a Ca^2+^ response confined to a spine head was observed. In all, 600-ms line scans of the active spine were then recorded while a dual stimulus (50-ms interstimulus interval) was delivered. Scans were repeated once every 30 s; a minimum of 15 trials in each condition were used to assess the release probability at the imaged synapse.

### Pharmacological manipulations

All recordings (except for the field potential recordings of heterosynaptic depression) were done in the presence of DL-2-amino-5-phosphonopentanoic (DL-APV; 100 μM), picrotoxin (100 μM) and (2*S*)-3-[[(1*S*)-1-(3,4-dichlorophenyl)ethyl]amino-2-hydroxypropyl](phenylmethyl) phosphinic acid (CGP55845; 5 μM) to block NMDA-, GABA_A_- and GABA_B_-receptors respectively. (1-[2-[*tris*(4-methoxyphenyl)methoxy]ethyl]-(*S*)-3- piperidinecarboxylic acid ((s)-SNAP5114; 100 μM), 1-(4,4-diphenyl-3-butenyl)-3-piperi dinecarboxylic acid (SKF89976A; 30 μM), DPCPX (10 μM), 2-[2-[4-(4-nitrobenzyloxy)phenyl]ethyl]isothiourea mesylate (KB-R7943; 50 μM), (±)-1-aminocyclopentane-*trans*-1,3-dicarboxylic acid (t-ACPD; 50 μM) were added to the perfusion solution. All above drugs were from Tocris Bioscience (Bristol, UK). β-alanine (1 or 2 mM), FAC (5 mM; minimum 30 min of pre-incubation) and γ-Aminobutyric acid (GABA; 5 or 30 μM) were purchased from Sigma-Aldrich (Dorset, UK).

### Statistical analysis

Data were analysed using GraphPad Prism software (GraphPad software, San Diego CA, USA) or SPSS (SPSS Inc., USA) and are presented as mean±s.e.m. Imaging and electrophysiological data are available upon request. Statistical analysis was performed using two-tailed paired or unpaired Student's *t*-test and ANOVA as detailed. Normality was tested using Shapiro-Wilk test. Differences were considered as significant at *P*<0.05, and this was corrected for multiple comparisons using the Holm-Bonferroni correction.

### Data availability

The data that support the findings of this study are available from the corresponding authors upon request.

## Additional information

**How to cite this article**: Boddum, K. *et al*. Astrocytic GABA transporter activity modulates excitatory neurotransmission. *Nat. Commun.* **7**, 13572 doi: 10.1038/ncomms13572 (2016).

**Publisher's note**: Springer Nature remains neutral with regard to jurisdictional claims in published maps and institutional affiliations.

## Supplementary Material

Supplementary Information Supplementary Figures 1 - 3
