# Supplementary material for: Astrocytic GABA transporter activity modulates excitatory neurotransmission
Source: Nat Commun. 2016 Nov 25;7:13572. doi: 10.1038/ncomms13572 (PMC5133667; doi:10.1038/ncomms13572)
Supplement: Supplementary Information — Supplementary Figures 1 - 3 [file ncomms13572-s1.pdf]

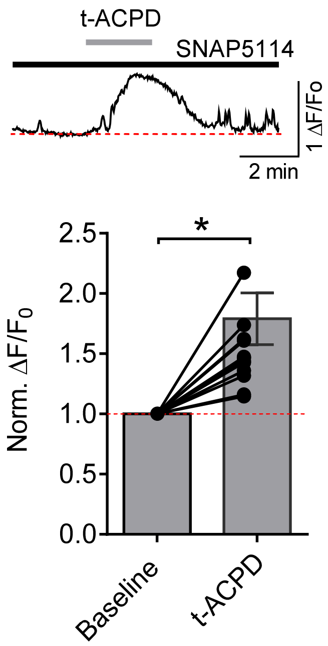

Supplementary Figure 1. Activation of metabotropic glutamate receptors induces Ca^{2+} increase in the presence of GAT-3 inhibitor. *Top*: representative trace showing change in astrocytic Ca^{2+} level following application of t-ACPD (50 μM) in the presence of SNAP5114. *Bottom*: Summary graph (n = 14 cells/2 animals); individual recordings represented by dots, means displayed by bars, error bars represent SEMs; * p = 0.003 (paired t-test compared to baseline).

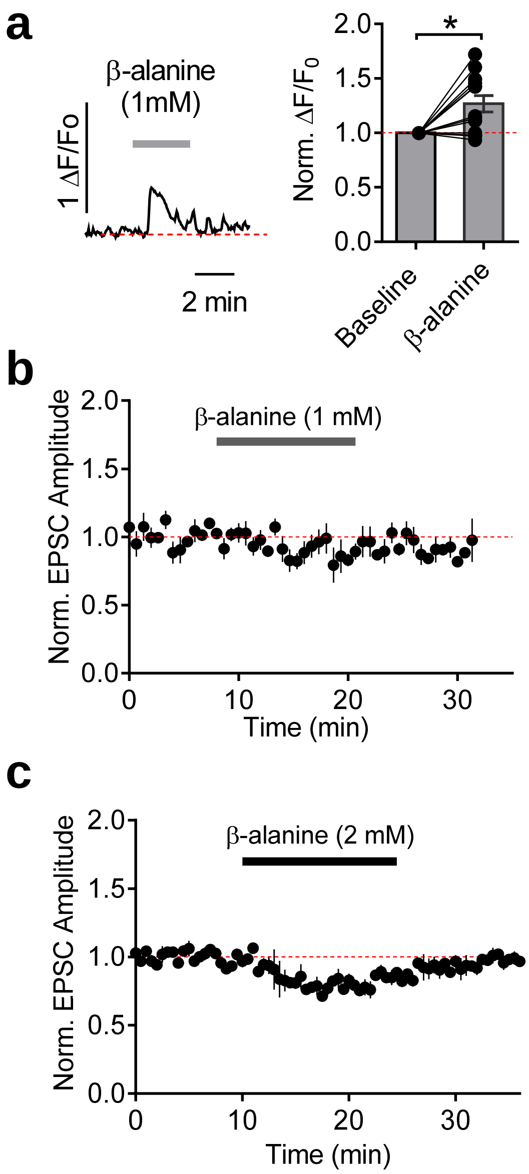

Supplementary Figure 2. β-alanine action on astrocytic Ca^{2+} responses and EPSCs. (**a**) β-alanine (1 mM) induces Ca^{2+} response in astrocytes (n = 13 cells/2 animals). Note that Ca^{2+} increase is smaller than that induced by 30 μM GABA (c.f. Fig. 4e). (**b**) Normalized EPSC amplitudes in response to application of 1 mM β-alanine (n = 6 cells/2 animals). (**c**) Normalized EPSC amplitudes in response to application of 2 mM β-alanine (n = 3 cells/2 animals). Error bars represent SEMs, * p = 0.004 (paired t-test compared to baseline).

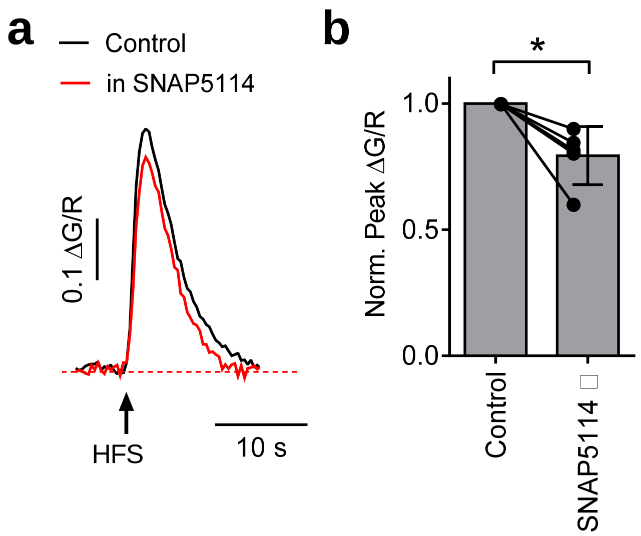

Supplementary Figure 3. Astrocytic Ca^{2+} responses to a one second, 100 Hz tetanus are reduced in the presence of the GAT-3 inhibitor SNAP5114 (100 µM). (**a**) Mean Ca^{2+} indicator fluorescence response (n = 5 cells/5 animals) to 100 Hz Schaffer collateral stimulation in control conditions (*black*) and in SNAP5114 (*red*). (**b**) Peak Ca^{2+} response amplitude for individual recordings (*dots*) and mean of five cells (*bars*). Error bars represent SEMs; * p = 0.016 (paired t-test).
